# O.2.3-9 The working shifts & physical literacy factors associated with physical activity level in various settings among nurses: preliminary results

**DOI:** 10.1093/eurpub/ckad133.135

**Published:** 2023-09-11

**Authors:** Nida Coşkun, Dilara Ebru Uçar, Günay Yildizer

**Affiliations:** Eskisehir Technical University, Turkiye; Anadolu University, Turkiye; nida10coskun10@gmail.com; Eskisehir Technical University, Turkiye

## Abstract

**Purpose:**

This study aims to examine (I) the importance of working shifts on physical activity (PA) level and (II) the association between physical literacy (PL) and energy spent with PA in occupational, leisure-time, and sports settings among nurses who live most of their time in healthcare environments and are aware of health literacy and PA.

**Methods:**

The data of this cross-sectional study were collected from 201 nurses (M_AGE_= 34.53±8.41; M_BMI_= 24.32±4.17) nurses working at hospitals in Eskisehir. Woking shifts were 08:00-16:00 (n = 53), 16:00-08:00 (n = 19), alternating between 08:00-16:00 and 16:00-08:00 (n = 47), and 24-hour shifts with a day off between shifts (n = 82). The 7-Day PA Assessment Questionnaire developed by Karaca and Turnagöl (2007) was used as a data collection tool as it enables individuals to report their various types of physical activities (occupational, leisure-time, sport) in the past seven days, and the energy spent is computed in terms of MET. Moreover, the Turkish version of the PL Questionnaire (Munusturlar and Yıldızer, 2021) was used to measure PL level in knowledge, communication, and self-awareness subdimensions. The data were analyzed with One-way ANOVA and multiple linear regression models.

**Results:**

The one-way ANOVA results indicated nurses working in 24-hour shifts reported significantly higher MET values (276.05±71.07 MET/week) in occupational PA than nurses working between 08:00-16:00 (222.00±53.70 MET/week) and 16:00-08:00 (223.27±73.67 MET/week) shifts, F_(3, 200)_=8.539, p < 0.001. Regression models indicated that PL was only associated with spent energy in sports activities [F_(4,200)_=9.21, p < 0.001], but not with occupational and leisure-time PA. Lastly, knowledge was the only PL factor associated with energy spent on sports activities (ß=1.35, OR:0.77-1.93).

**Conclusions:**

24-hour working shifts with day-offs increase energy expenditure with occupational physical activity, however, there was no significant differences were found in energy expenditure with leisure time or sports activities in favor of those who work in these shifts. Moreover, increased physical literacy knowledge is the only factor for increasing physical activity participation through sports activities. Hence, policies, PA prescriptions, and interventions should focus on PL knowledge and understanding.

**Support/Funding Source:**

This study was supported by Eskişehir Technical University Scientific Research Projects Commission under grant no: 23LÖT069.

